# The effect of individual exercise rehabilitation program on ischemic burden and cardiac function in patients with ischemic non-obstructive coronary heart disease: a randomized parallel controlled clinical trial

**DOI:** 10.3389/fcvm.2025.1421923

**Published:** 2025-03-07

**Authors:** Yuan Wen, Yuanyuan Zhang, Qingquan Lv, Weiqun Lan, Yi Shu, Qiuhuan Qi, Hongping Hu, Othman Zakaria Saleh

**Affiliations:** ^1^ Department of Cardiovascular Medicine, Wuhan Hankou Hospital, Wuhan, China; ^2^ Department of Medical Affairs, Wuhan Hankou Hospital, Wuhan, China; ^3^ Department of Emergency Medicine, Wuhan Hankou Hospital, Wuhan, China; ^4^ Department of Cardiology, West China Hospital, Sichuan University, Chengdu, China

**Keywords:** individualized exercise rehabilitation program, ischemic non-Obstructive coronary heart disease, ischemic load, burden, heart

## Abstract

**Background:**

Coronary heart disease (CHD) is a pervasive chronic condition that poses a significant threat to global health and mortality worldwide. Given the severity of this disease, it is imperative to consider pivotal factors such as age, concurrent diseases, and physical frailty of patients diagnosed with ischemic non-obstructive CHD prior to initiating cardiopulmonary rehabilitation. Consequently, the objective of this study is to investigate the impact of an individualized exercise rehabilitation program, on the ischemic burden in patients suffering from ischemic non-obstructive CHD.

**Methods:**

From February 2019 to July 2021, a cohort of one hundred patients diagnosed with ischemic non-obstructive CHD were recruited and randomly allocated into two groups. The control group underwent a standard rehabilitation program, while the intervention group participated in an individualized exercise rehabilitation program. This program was tailored to each patient, with a 50% power intensity exercise prescription derived from the results of the patient's Cardiopulmonary Exercise Testing (CPET) evaluation. The therapeutic effect, total myocardial ischemic burden (TIB), the effective rate of TIB reduction, pulmonary function indices, cardiac function, and the incidence of adverse events compared between the two groups.

**Results:**

The intervention group demonstrated a higher effective rate. TIB in the intervention group was significantly reduced at the 1,2, and 4-week marks post-intervention and exhibited a higher effective rate of total myocardial ischemia load reduction. Post-intervention, there were improvements in the Forced Vital Capacity (FVC), Forced Expiratory Volume in one second (FEV1), and the FEV1/FVC ratio, with the intervention group showing higher values for these parameters. Cardiac function was enhanced following the intervention, with the intervention group displaying higher Left Ventricular Ejection Fraction (LVEF) and Carbon Monoxide (CO) levels, and a lower Left Ventricular End-Diastolic Dimension (LVEDD) compared to the control group (*P* < 0.05).

**Conclusion:**

An individualized exercise rehabilitation regimen for patients diagnosed with ischemic non-obstructive CHD can effectively compensate for the lack of physical activity. This regimen has demonstrated its ability to enhance the clinical therapeutic effect, reduce the total load of myocardial ischemia, improve pulmonary function indices and cardiac function, and decrease the incidence of cardiovascular adverse events.

**Clinical Trial Registration:**

identifier (TJ-IRB20210716).

## Introduction

Coronary heart disease (CHD) is a leading cause of morbidity and mortality worldwide, responsible for approximately 30% of global deaths (1). In China, the incidence of coronary heart disease (CHD) was 111.92 per 100,000 people in 2017 (2). Despite a 50% reduction in age-standardized mortality over the past 30 years, the proportion of younger individuals affected by CHD has increased (3). The aging population, projected to reach 37.8% by 2050, will significantly contribute to the rise in CHD cases, especially among the elderly (4, 5). In the United States, acute coronary events occur every 25 s, leading to one death every minute (1, 6). Advances in diagnostic methods, such as coronary angiography (CAG), have significantly improved the detection of CHD. However, while obstructive CHD is defined by a coronary artery stenosis of 50% or greater, non-obstructive CHD (NOCAD) presents a distinct clinical challenge. NOCAD patients exhibit symptoms of myocardial ischemia (7).

Non-obstructive CHD is particularly prevalent in women and is associated with a higher risk of myocardial ischemia despite the absence of overt coronary obstruction (8, 9).

Myocardial ischemia, resulting from an imbalance between the myocardial oxygen supply and demand, is the hallmark of CHD. This condition impairs myocardial energy metabolism and affects normal heart function (10). Given the increasing prevalence of myocardial ischemia, particularly in middle-aged and elderly populations, it is critical to focus on both the prevention and treatment of ischemic conditions, including non-obstructive CHD (11). Despite the use of pharmacological interventions to manage risk factors like hypertension, diabetes, and dyslipidemia, these measures often prove insufficient in reducing ischemic burden and improving outcomes (12).

Recent studies have highlighted the importance of exercise rehabilitation as a vital intervention for patients with CHD, including those with non-obstructive disease. Cardiopulmonary exercise rehabilitation has been shown to improve physical function, enhance cardiopulmonary fitness, and reduce cardiovascular event rates (9, 11).

While addressing the chronic nature of CHD remains a challenge, especially in hospital settings, home-based cardiopulmonary rehabilitation (CPR) has gained increasing importance. It allows for self-management, enhances patient autonomy, and improves quality of life through accessible, cost-effective methods (13). Despite early efforts, by 2012, only eight CPR centers existed, highlighting challenges in expanding services (14). However, with the adoption of Western models and the “Exercise is Medicine” philosophy, CPR for CHD has gained more attention (15). Despite numerous guidelines for CPR, many patients, especially the elderly with comorbidities, fail to achieve optimal outcomes. Notably, there is a gap in studies on age-related conditions, such as weakness, during CPR (16). Individualized exercise rehabilitation programs have emerged to address these issues, particularly for patients with ischemic non-obstructive CHD. These programs improve recovery by enhancing exercise tolerance and reducing adverse outcomes, helping to overcome gaps in self-care knowledge and standardization (17).

Exercise rehabilitation programs that focus on enhancing cardiopulmonary function, muscle strength, flexibility, and coordination may hold promise for improving outcomes in patients with ischemic non-obstructive CHD. Recent findings suggest that tailored rehabilitation programs designed to address the patient's frailty, comorbidities, and exercise capacity could provide greater benefits than traditional, one-size-fits-all approaches (18). Importantly, exercise has the potential to reduce ischemic burden, mitigate myocardial ischemia, and improve cardiac function, offering a holistic approach to managing this condition.

Exercise rehabilitation has emerged as a promising intervention for improving outcomes in patients with ischemic heart disease, including those with non-obstructive coronary artery disease (NOCAD). Several studies have highlighted the efficacy of exercise-based rehabilitation in reducing ischemic burden and improving cardiovascular outcomes. A systematic review and meta-analysis found that exercise training significantly improves physical function and reduces cardiovascular event rates in patients with ischemic heart disease, including those with non-obstructive coronary disease (19). Additionally, a study by Kissel et al. (20) reported that exercise rehabilitation in NOCAD patients not only enhanced cardiopulmonary fitness but also contributed to a reduction in the ischemic burden, leading to better overall heart function (20). Moreover, evidence from randomized trials underscores the effectiveness of cardiac rehabilitation in enhancing quality of life and reducing mortality rates in ischemic heart disease patients, including those with NOCAD (21). While the majority of NOCAD patients do not achieve optimal benefits from traditional rehabilitation programs, personalized exercise interventions have been shown to offer greater improvements by addressing individual frailty, comorbidities, and exercise capacity (22). Therefore, the integration of individualized exercise rehabilitation into the management of NOCAD represents a critical strategy for improving prognosis and reducing ischemic load.

Given the growing recognition of the benefits of exercise rehabilitation, this study aims to assess the impact of an individualized exercise rehabilitation program on ischemic burden and cardiac function in patients with ischemic non-obstructive CHD. This study seeks to enhance the understanding of how tailored exercise interventions can improve outcomes in this patient group.

## Methods

### Study design and setting

The present study is a randomized parallel controlled clinical trial which conducted from February 2019 to July 2021, in Wuhan Hankou Hospital, China.

### Participants and sampling

The patients were randomly selected from among the people who visited the medical centers and met the inclusion criteria. The randomization process was done using a table of random numbers, which assigned the patients to two sports rehabilitation groups or the control group.

Inclusion criteria: (1) Eligible patients included those who had been diagnosed with non-obstructive ischemic CHD based on the previous angiography they had performed, or patients who had a history of being discharged from the hospital with this diagnosis (23, 24). (2) Patients whose condition has stabilized following standardized drug treatment, with no apparent clinical symptoms, and whose cardiac function, as classified by the NYHA functional was grade Ⅰ and Ⅱ (grade Ⅰ: No limitation of physical activity. Ordinary physical activity does not cause symptoms of HF and grade Ⅱ: Slight limitation of physical activity. Comfortable at rest, but ordinary physical activity results in symptoms of HF) (25).

Exclusion criteria: (1) Patients with lower limb dyskinesia, paralysis, or other motor difficulties that preclude testing or training; (2) Patients with NYHA cardiac function grades III or IV, or other severe activity limitations (grades III: Marked limitation of physical activity. Comfortable at rest, but less than ordinary activity causes symptoms of HF and grades IV: Unable to carry on any physical activity without symptoms of HF, or symptoms of HF at rest; (3) Patients with complications from severe systemic diseases such as unstable angina pectoris, acute myocardial infarction, malignant arrhythmia, and hepatic and renal dysfunction (25–27); (4) Patients were advised to maintain their usual eating habits and avoid significant changes in their diet during the study period and any of the patients who made significant changes in their usual diet (such as starting a special diet such as vegetarianism, ketogenic, etc.) would be excluded from the study.

**Figure 1:**
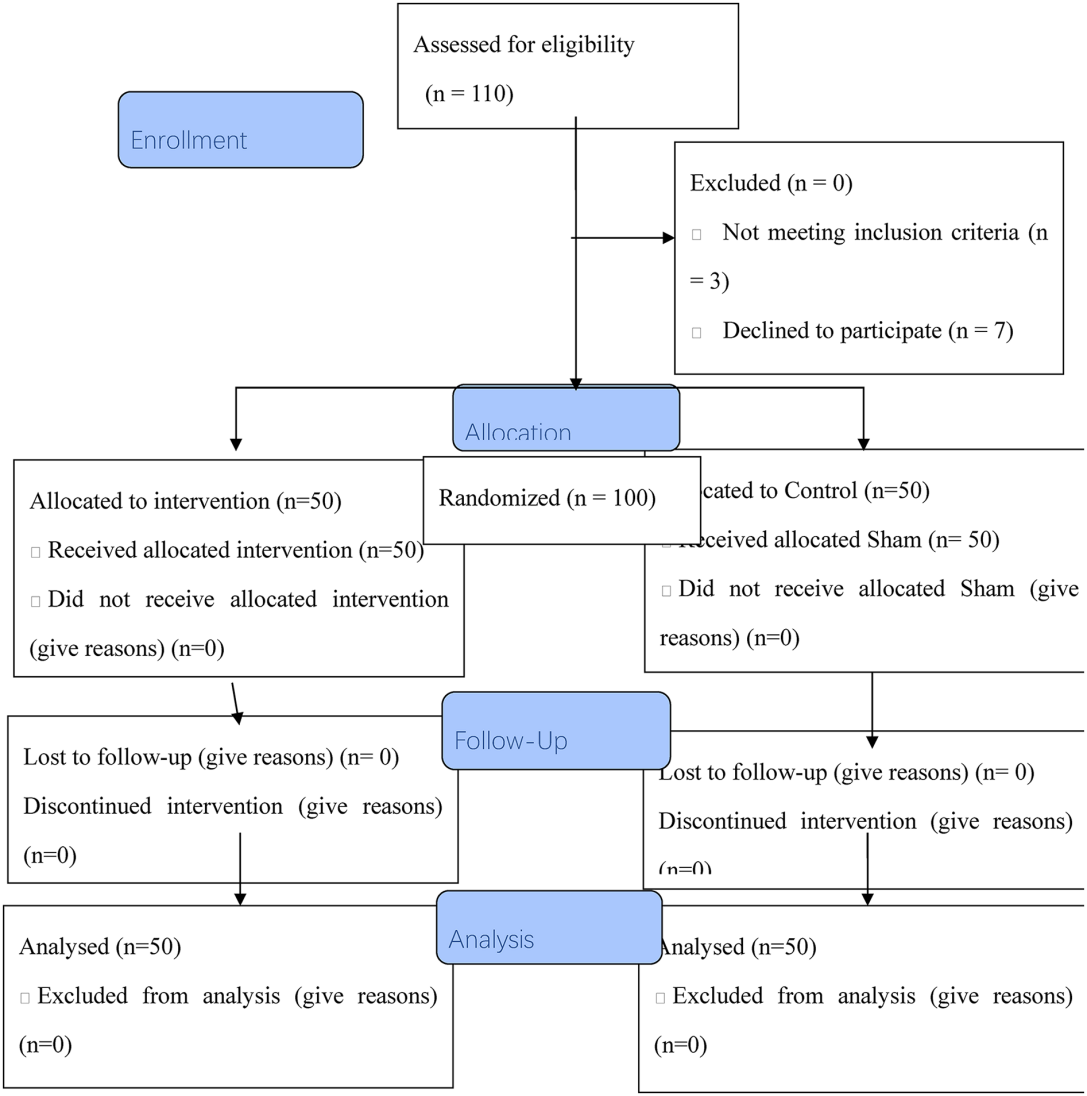
The flow chart of the study.

Power analysis calculations with G*Power software indicate that (power = 80%, *P* = 0.05, number of groups = 2, and number of measurements = 2) 100 participants would be needed to detect an effect size of 0.55. Totally, 110 samples were assessed for eligibility, of which, 100 eligible participants allocated to the two groups. Finally, all 100 participants finished the study (Figure 1).

### Treatment methods

In the control group, the amount of activity is designed according to the AHA (28) guideline, which the control group underwent a routine rehabilitation program, which included aerobic exercise 5–7 days a week, starting with 10 min each session and gradually increasing to 30 min and it was done for 8 weeks. According to the AHA (28) the exercises were primarily of low to medium intensity, involving large muscle groups in slow movements such as aerobics, that includes such as practicing Qigong (29), Taijiquan (29), walking, fitness running, outings, and fishing. Resistance exercises were performed 2–3 days a week at low to medium intensity, with a focus on avoiding strain. Flexibility exercises, balance exercises, and other functional exercises were conducted 2–3 times a week. Additionally, a lifestyle of increased physical activity and reduced sedentary behavior was encouraged.

Qigong is a versatile form of exercise that can be practiced anywhere, at any time, without the need for specialized equipment, and with minimal time commitment. As a result, it can easily be incorporated into daily routines and integrated into a comprehensive cardiac rehabilitation program. Qigong is an ancient Chinese martial art that encompasses a variety of exercises, such as Dao-Yin-Shu (physical and breathing exercises), Wu-Qin-Xi (five animals play), Baduanjin (eight silken movements), and Yi-Jin-Jing (changing tendons exercises). Tai Chi is a well-known practice that evolved from the Qigong tradition. Tai Chi and Qigong practices (TQPs) generally involve slow, deliberate movements synchronized with meditation and controlled breathing techniques. Moreover, all TQPs are based on the principle that any form of Qigong helps cultivate balance and harmonize vital energy (qi), functioning as a holistic, interconnected, and interactive system (29).

According to the guidelines of the American Heart Association (AHA), at least 300 min of exercise in patients with heart problems can have more benefits and relief (28).

Therefore, in addition to the exercises performed by the control group, the intervention group was also subjected to an individual sports rehabilitation program.

This program was designed based on each patient's own Cardiopulmonary Exercise Testing (CPET) evaluation (Version 7, ergostik, Geratherm, Bad Kissingen, Germany) (30, 31). The individual exercise rehabilitation program effected on ischemic load in patients with ischemic non-obstructive CHD. The exercise intensity was set at 50% power, calculated with the following formula:

$$\frac{\left(ATMP-PIR\times 0.75\right)}{2}+\frac{\;(EEMP-PIR\times 0.75)}{2}$$

(Where ATMP is anaerobic threshold measured power, PIR is power increasing rate, and EEMP is extreme exercise measured power). The exercise duration was 30 min per day, supplemented by a 5 min warm-up (no power load) and a 5 min recovery exercise (no power load), totaling 40 min. The exercise frequency was 5 days per week over an 8-week cycle. The treadmill speed was maintained at 60 r/min during training. Throughout the exercise rehabilitation process, patients' blood pressure and pulse were monitored, and any changes in their condition were closely observed.

### Observation indices

#### Measures

##### Curative effect evaluation

Upon completion of the rehabilitation exercise intervention, the therapeutic effect was assessed. The evaluation involved the following cardiac function indices: Left Ventricular Ejection Fraction (LVEF), Cardiac Output (CO), and Left Ventricular End-Diastolic Dimension (LVEDD); and pulmonary function indices: Forced Vital Capacity (FVC), Forced Expiratory Volume in one second (FEV1), and the FEV1/FVC ratio (10). The outcomes were categorized as follows:

• Markedly Effective: The improvement in cardiac function index and pulmonary function index exceeded 70%, indicating a remarkable enhancement in the patient's heart and lung function.
• Effective: The improvement in cardiac function index and pulmonary function index ranged between 50% and 69%, signifying an enhancement in the patient's heart and lung function.
• Ineffective: The improvement in cardiac function index and pulmonary function index was less than 50%, suggesting no enhancement in the patient's heart and lung function.

The total effective rate = sum of the markedly effective rate + the effective rate.

##### Total myocardial ischemic load

A 24 h ambulatory electrocardiogram examination was conducted prior to the intervention, and subsequently at 1 week, 2 weeks, and 4 weeks post-intervention. The TIB was evaluated at each of these time points.

##### Total load efficiency of myocardial ischemia

The TIB, as measured by a 24 h dynamic electrocardiogram, was evaluated before and after treatment. The unit of measurement was (mm · min)/24 h. The effectiveness of the treatment was determined based on the decrease in the detection value. If the detection value decreased by more than 40 (mm · min)/24 h, it was recorded as markedly effective. If the decrease was between 10 and 40 (mm · min)/24 h, it was recorded as effective. If the decrease was less than 10 (mm · min)/24 h, it was recorded as ineffective. The total effective rate was calculated as the sum of the markedly effective rate and the effective rate.

##### Pulmonary function index

Pulmonary function tests were conducted before and after the intervention, with indices such as FVC, FEV1, and the FEV1/FVC ratio recorded pre- and post-intervention.

##### Cardiac function index

Cardiac function changes, including LVEF, CO, and LVEDD, were measured using color Doppler ultrasound before and after the intervention.

##### Occurrence of adverse events

Statistics of adverse events were compiled, which included heart failure, cerebral infarction, and myocardial infarction.

All cardiac measurements were taken at the same time point for all patients, specifically at baseline and after the intervention.

### Statistical analysis

The data were analyzed using SPSS 21.0 (International Business Machines Corporation, Chicago, Illinois, USA). The measurement data were represented as mean and standard deviation. A two-independent sample *t*-test was utilized for comparison between groups, while a paired sample *t*-test was used for within-group comparisons pre- and post-treatment. Counting data were represented by frequency and percentage, and analyzed using either the Fisher exact probability test or the *Chi square* test. Repeated measurement data were analyzed using repeated measures ANOVA, with post-hoc testing conducted using the Student-Newman-Keuls method. A significance level of α = 0.05 (*P* < 0.05) was adopted, indicating that statistically significant differences were found.

## Results

The control group consisted of patients aged between 43 and 74 years, with a mean age of 65.53 ± 3.55 years, including 19 males and 31 females. The intervention group comprised patients aged between 44 and 76 years, with a mean age of 65.57 ± 3.45 years, including 20 males and 30 females. Overall, the data did not exhibit any statistical significance (*P* > 0.05) (Table 1).

**Table 1:** Comparison the characteristics of the participants between the two groups.

Group	*N*	Age (Mean ± SD)	Sex (*n*/%)	Cardiac function (*n*/%)
Control	50	65.53 ± 3.55	Male: 19/38, Female: 31/62	Grade *Ι*: 25/50 Grade *Π*: 25/50
Intervention	50	65.57 ± 3.45	Male: 20/40, Female: 30/60	Grade *Ι*: 24/48 Grade *Π*: 26/52
*t*/*χ*^2^ value		0.054	0.090	0.005
*P*		0.85	0.956	>0.99

The intervention was extremely effective in 45 patients, effective in 4 patients, and ineffective in 1 patient, the total effective rate was 98.00%; the routine rehabilitation program was extremely effective in 34 patients, effective in 10 patients, ineffective in 6 patients, and the effective rate was 88.00%. The research group had a higher effective rate (*P* = 0.011).

The total ischemia burden (TIB) in the intervention group was found to be significantly lower at 1-week, 2-weeks, and 4-weeks post-intervention (*P* < 0.05), as illustrated in Table 2.

**Table 2:** Comparison of total myocardial load before and after intervention.

Group	*N*	Before	After 1 week	After 2 weeks	After 4 weeks
Control	50	61.94 ± 3.31	56.14 ± 3.31	50.83 ± 3.31	46.93 ± 3.31
Intervention	50	61.55 ± 3.45	45.82 ± 1.23	40.96 ± 3.43	31.86 ± 2.44
F value		119.179	119.179	119.179	119.179
*P*		>0.05	<0.001	<0.001	<0.001

Values and unit: [x¯ ± s, mm · min/24 h].

In the research group, the intervention was found to be extremely effective in 28 patients, effective in 20 patients, and ineffective in 2 patients, yielding a total effective rate of 96.00%. In contrast, the control group had 11 patients where the intervention was extremely effective, 20 patients where it was effective, and 19 patients where it was ineffective, resulting in an effective rate of 62.0%. The intervention group demonstrated a significantly higher effective rate of myocardial ischemia (*P* < 0.05).

### Comparison of pulmonary function indexes

Post-intervention, there was an enhancement in the FVC, FEV1, and the FEV1/FVC ratio. Furthermore, the intervention group demonstrated higher values for FVC, FEV1, and FEV1/FVC (*P* < 0.05) (Table 3).

**Table 3:** Comparison of pulmonary function indexes before and after intervention.

Group	FVC (L)		FEV_1_ (L)		FEV_1_/FVC (%)	
	Before	After	Before	After	Before	After
Control	2.48 ± 0.31	2.54 ± 0.42	2.09 ± 0.33	2.18 ± 0.22	78.93 ± 4.31	80.49 ± 1.22
Intervention	2.50 ± 0.23	2.69 ± 0.12	2.10 ± 0.32	2.20 ± 0.12	78.45 ± 3.56	85.91 ± 3.12
Independent *t*-test	0.366	4.047	0.153	5.361	0.607	11.440
*P*	0.784	0.002	0.626	0.006	0.548	0.005

### Comparison of cardiac function indexes

Following the intervention, there was an enhancement in cardiac function. The intervention group demonstrated higher values for LVEF and CO and a lower value for LVEDD (*P* < 0.05) (Table 4).

**Table 4:** Comparison of cardiac function indexes before and after intervention.

Group	LVEF (%)		CO (L/min)		LVEDD (mm)	
	Before	After	Before	After	Before	After
Control	42.56 ± 3.21	50.92 ± 3.36	4.39 ± 0.31	5.22 ± 1.33	67.97 ± 3.31	56.96 ± 3.31
Intervention	42.69 ± 3.31	57.91 ± 3.44	4.38 ± 0.55	5.85 ± 1.56	67.98 ± 3.44	52.96 ± 4.46
Independent *t-*test	0.199	10.278	0.111	2.173	0.014	5.092
*P*	1.025	0.009	1.924	0.006	2.814	0.004

### Comparison of the incidence of adverse events

In the intervention group, there were no instances of cardiac failure, one instance of cerebral infarction, and one instance of myocardial infarction. This resulted in a total incidence rate of 4.00%. In contrast, the control group had two instances of cardiac failure, three instances of cerebral infarction, and three instances of myocardial infarction, leading to a total incidence rate of 16.00%. The data did not exhibit any notable differences (*χ*^2^ = 4.0, *P* = 0.055).

## Discussion

This study aimed to evaluate the impact of an individualized exercise rehabilitation program on ischemic burden and cardiac function in patients with ischemic non-obstructive coronary artery disease (NOCAD). The results demonstrated that the individualized exercise rehabilitation program significantly outperformed the standard rehabilitation program in terms of reducing ischemic burden and improving both cardiac and pulmonary function. Additionally, the intervention group showed a better overall improvement in clinical outcomes, including a lower incidence of adverse events. The following sections provide a detailed discussion of these findings, comparing them with relevant studies and interpreting the results in the context of current knowledge.

### Effectiveness of the individualized exercise rehabilitation program

The results showed that the individualized exercise rehabilitation program was significantly more effective than the standard rehabilitation program. In the intervention group, 45 patients showed a complete response (with an effectiveness rate of 98%), while in the control group, 34 patients showed a complete response, yielding an overall effectiveness rate of 88%. This difference was statistically significant (*P* = 0.011). These findings clearly indicate that personalized exercise rehabilitation programs, which are tailored to the individual needs of patients, are more effective than standard programs. This is consistent with previous studies that have emphasized the benefits of personalized rehabilitation in improving clinical outcomes (19, 20). Particularly in NOCAD patients, where the absence of overt coronary artery obstruction complicates management, a personalized approach to rehabilitation seems to offer substantial benefits.

### Reduction in myocardial ischemic burden

One of the key findings in this study was the significant reduction in total ischemic burden (TIB) in the intervention group. At one, two-, and four-weeks post-intervention, the ischemic burden in the intervention group decreased significantly (*P* < 0.001). This reduction in ischemic burden is a clear indication of the positive effect of the individualized exercise rehabilitation program in restoring the balance between myocardial oxygen demand and supply. These results align with previous studies that reported the beneficial effects of exercise rehabilitation on reducing ischemic burden in patients with ischemic heart disease (21). For instance, the study by Kissel et al. (20) showed that exercise training not only reduced ischemic burden but also improved overall cardiac function in patients with NOCAD (20). The reduction in ischemic burden in this study can likely be attributed to improved physical fitness, increased coronary blood flow, and better cardiopulmonary function, which collectively support better myocardial oxygen supply during exercise and daily activities. A study by Ninghong et al. (32) demonstrated that rehabilitation training interventions for elderly patients with coronary artery disease can enhance cardiopulmonary function, improve quality of life, and effectively alleviate patients' adverse psychological conditions.

### Improvement in cardiac function

The study also found significant improvements in cardiac function in the intervention group. The left ventricular ejection fraction (LVEF) and cardiac output (CO) both increased, while the left ventricular end-diastolic diameter (LVEDD) decreased in the intervention group (*P* < 0.05). Specifically, LVEF improved from 42.69 ± 3.31% to 57.91 ± 3.44%, and CO increased from 4.38 ± 0.55 L/min to 5.85 ± 1.56 L/min (*P* = 0.006 and *P* = 0.009, respectively). In contrast, there were no significant changes in these parameters in the control group. These findings suggest that the individualized exercise rehabilitation program had a notable impact on improving cardiac function. This is consistent with the findings of other studies, which have shown that exercise rehabilitation can improve heart function and reduce the risks associated with ischemic heart disease (21). In particular, NOCAD patients, who often experience subtle ischemic episodes despite the absence of severe coronary stenosis, can benefit from rehabilitation programs that enhance cardiac function and overall cardiovascular health.

### Improvement in pulmonary function

The intervention group also demonstrated significant improvements in pulmonary function. Specifically, the forced vital capacity (FVC), forced expiratory volume in one second (FEV1), and the FEV1/FVC ratio all improved post-intervention. In particular, the FEV1/FVC ratio in the intervention group increased from 78.45 ± 3.56% to 85.91 ± 3.12% (*P* = 0.005). This improvement in pulmonary function is a significant finding, as better pulmonary function supports better overall cardiovascular health by improving oxygen delivery to tissues during physical activity. These findings are consistent with other studies that have shown the benefits of exercise rehabilitation on respiratory function, especially in older patients or those with comorbid pulmonary conditions (20). Improving pulmonary function not only aids in daily physical activity but also reduces symptoms like dyspnea, further enhancing quality of life for NOCAD patients.

### Reduction in adverse event incidence

The incidence of adverse events was lower in the intervention group compared to the control group. In the intervention group, there was a 4% incidence rate of adverse events, which included one case of myocardial infarction and one case of cerebral infarction. In contrast, the control group had a 16% incidence rate, with two cases of cardiac failure, three cases of cerebral infarction, and three cases of myocardial infarction. Although the difference in adverse event rates was not statistically significant (*P* = 0.055), the clinical significance of this difference cannot be overlooked. The lower rate of adverse events in the intervention group suggests that the exercise rehabilitation program may help prevent some of the complications commonly associated with ischemic heart disease. These findings are in line with other studies that have highlighted the role of exercise-based rehabilitation in reducing adverse cardiovascular events and improving long-term outcomes for patients with ischemic heart disease (9, 21). One of the major challenges in implementing cardiac rehabilitation (CR) programs is the various barriers that may arise in practice. These include logistical issues, patient adherence, and the high cost of rehabilitation services. The cost-effectiveness of these programs is a critical issue in recent research, as the economic sustainability of CR programs is crucial for healthcare systems with limited resources. While the initial costs of implementing CR programs may be high, these costs are offset in the long term by reduced hospital admissions, decreased mortality rates, and improved quality of life for patients. For example, a study by Su et al. (33) demonstrated that exercise-based rehabilitation programs for patients with coronary heart disease (CHD) are cost-effective and can contribute to lowering long-term treatment costs. However, further research is needed to evaluate the precise economic impact of these programs in various clinical and geographical settings (33).

### Limitations and future research directions

Despite the promising results, there are several limitations in this study that should be considered. First, the sample size was relatively small, which may limit the generalizability of the findings. Larger studies with more diverse populations are needed to confirm the effectiveness of individualized exercise rehabilitation programs. Additionally, this study primarily focused on the short-term effects of the intervention. Future research should investigate the long-term effects of exercise rehabilitation on disease progression, quality of life, and mortality in NOCAD patients. Longitudinal studies would also help to better understand the sustainability of the observed improvements and the potential for relapse or further cardiovascular events. Furthermore, the use of total ischemic burden (TIB) as a measure of ischemia has been identified as a limitation. The unreliability of TIB compared to newer techniques (such as cardiac MRI or QCA) should be considered, and future studies should consider employing more advanced and reliable diagnostic tools to better assess ischemia. Finally, although we tried to check the most related clinical characteristics for the included participants and participants were randomly allocated to each group, some other variables including the history of chronic disease and the history of hospitalization have not been assessed. Such these variables may have confounding effect on the results. Therefore, it is suggested to be considered in the future studies.

## Conclusion

In conclusion, this study demonstrates that individualized exercise rehabilitation is a highly effective intervention for improving outcomes in patients with ischemic non-obstructive coronary artery disease. The program significantly reduced ischemic burden, improved both cardiac and pulmonary function, and decreased the incidence of adverse cardiovascular events. These findings align with previous research and emphasize the importance of personalized rehabilitation strategies for managing ischemic heart disease, particularly in patients with non-obstructive coronary artery disease. Given the growing body of evidence supporting the benefits of exercise rehabilitation, it is recommended that personalized exercise programs be integrated into the routine management of NOCAD patients as part of a comprehensive approach to improving cardiovascular health.

## Data Availability

The raw data supporting the conclusions of this article will be made available by the authors, without undue reservation.
